# Function of a mutant ryanodine receptor (T4709M) linked to congenital myopathy

**DOI:** 10.1038/s41598-023-41801-2

**Published:** 2023-09-05

**Authors:** Zsuzsanna É. Magyar, Judit Hevesi, Linda Groom, Robert T. Dirksen, János Almássy

**Affiliations:** 1 Department of Physiology, Faculty of Medicine, University of Debrecen, Debrecen, Hungary; 2 Department of Orthodontics, Faculty of Dentistry, University of Debrecen, Debrecen, Hungary; 3 Doctoral School of Molecular Medicine, University of Debrecen, Debrecen, Hungary; 4 Department of Pharmacology and Physiology, University of Rochester Medical Center, Rochester, NY USA; 5 Department of Physiology, Semmelweis University, Budapest, Hungary

**Keywords:** Pathogenesis, Physiology, Biophysics, Permeation and transport, Mechanisms of disease

## Abstract

Physiological muscle contraction requires an intact ligand gating mechanism of the ryanodine receptor 1 (RyR1), the Ca^2+^-release channel of the sarcoplasmic reticulum. Some mutations impair the gating and thus cause muscle disease. The RyR1 mutation T4706M is linked to a myopathy characterized by muscle weakness. Although, low expression of the T4706M RyR1 protein can explain in part the symptoms, little is known about the function RyR1 channels with this mutation. In order to learn whether this mutation alters channel function in a manner that can account for the observed symptoms, we examined RyR1 channels isolated from mice homozygous for the T4709M (TM) mutation at the single channel level. Ligands, including Ca^2+^, ATP, Mg^2+^ and the RyR inhibitor dantrolene were tested. The full conductance of the TM channel was the same as that of wild type (wt) channels and a population of partial open (subconductive) states were not observed. However, two unique sub-populations of TM RyRs were identified. One half of the TM channels exhibited high open probability at low (100 nM) and high (50 μM) cytoplasmic [Ca^2+^], resulting in Ca^2+^-insensitive, constitutively high P_o_ channels. The rest of the TM channels exhibited significantly lower activity within the physiologically relevant range of cytoplasmic [Ca^2+^], compared to wt. TM channels retained normal Mg^2+^ block, modulation by ATP, and inhibition by dantrolene. Together, these results suggest that the TM mutation results in a combination of primary and secondary RyR1 dysfunctions that contribute to disease pathogenesis.

## Introduction

Ryanodine receptor type 1 (RyR1) is the Ca^2+^ channel of the sarcoplasmic reticulum (SR) of the skeletal muscle. When RyR1 channels open under the control of an action potential in the sarcolemma, Ca^2+^ is released from the SR, which is used to drive muscle contraction. The communication between the sarcolemma and RyR1 is mediated by the dihydropyridine receptor (DHPR) in a voltage dependent manner through an allosteric mechanism, called excitation–contraction coupling (ECC)^1, 2^. RyR1 channels are regulated by a variety of ligands, including Ca^2+^, ATP and Mg^2+^^3–9^. In terms of regulation, the most relevant ligand is Ca^2+^, which activates RyR1 in the micromolar range and allows maximal channel activity at 50 µM^6^. The role of cytoplasmic Ca^2+^ in ECC is to open RyR channels that are not directly allosterically coupled to DHPRs, within a process termed Ca^2+^-induced Ca^2+^ release^10, 11^. In this way Ca^2+^ release amplifies the Ca^2+^ signal. Mg^2+^ inhibits RyR1 channels in the millimolar range by competing for the Ca^2+^ binding site^8^. ATP is one of the most effective agonists of RyR1, as it significantly activates the channel even in the absence of Ca^2+^ and can further enhance RyR1 channel open probability even in 50 µM Ca^2+^^9^.

Mutations impairing RyR function or its regulation cause a wide spectrum of muscle disorders, collectively called ryanopathies^12^. Among these, RyR1-related myopathies are often classified according to their specific histological presentation. Major histopathological groups of RYR1-related myopathies include central core disease (CCD), multiminicore disease, core-rod myopathy, and centronuclear myopathy. The patients suffer from mild, slowly- or non-progressive disabilities due to proximal muscle weakness and fatigue, low muscle tone and slow contraction. Symptoms also include respiratory muscle weakness, scoliosis, orthopedic deformities, pronounced facial weakness and dysmorphism, ptosis, ophthalmoparesis, exertional heat stroke, exertional myalgia and malignant hyperthermia susceptibility^13, 14^. Malignant hyperthermia is an idiosyncratic reaction to volatile anaesthetics (such as halothane and isoflurane) and succinylcholine. Susceptible patients exhibit muscle contractures all over the body when exposed to therapeutic concentrations of these trigger-drugs. Consequently, the body temperature rapidly increases, acidosis and hyperkalaemia develop, leading to an acute life-threatening condition unless the body is cooled down and dantrolene, an RyR1 inhibitor and muscle relaxant, is immediately administered^15–20^.

At the cellular level, RyR1 channels carrying certain MH causing mutations showed elevated basal activity when expressed in skeletal muscle fibres, indicating that there was a resting Ca^2+^ leak from the SR. Nevertheless, both the Ca^2+^ content of the SR and the resting myoplasmic Ca^2+^ concentration remained normal, indicating that the leak was low enough to be compensated by the SR Ca^2+^ pump. However, the threshold for activation of Ca^2+^ release by voltage and caffeine was significantly lower, suggesting that the mutations increased the sensitivity of RyR1 to activation by both endogenous (i.e. DHPR electromechanical uncoupling) and exogenous (caffeine) activators^21–23^. In contrast, RyR1 channels carrying other more severe CCD mutations showed a substantial elevation resting activity, leading to decompensated Ca^2+^ leak and consequent depletion of Ca^2+^ from the SR, resulting in attenuated Ca^2+^ release^23, 24^. Other mutations located within the RyR1 pore region result in a reduction in Ca^2+^ RyR1 permeation, and thus, a function uncoupling of excitation from SR Ca^2+^ release (termed “EC uncoupling”)^23, 25^. Apparently, the clinical symptoms can be explained by both of these mechanistic alterations in RyR1 function (RyR1 Ca^2+^ leak and EC uncoupling).

In some new cases of RyR1-related myopathies, pathologic SR Ca^2+^ leak was shown to be associated with low levels of the regulatory protein FKBP12 (calstabin) bound to the RyR1^26^. FKBP12 stabilizes the closed state of the RyR1 channel and dissociation causes leaky RyR1 channels by promoting subconductive openings. Posttranslational RyR1 modifications, such as PKA mediated hyperphosphorylation, oxidation and S-nitrosylation were reported to dissociate FKBP12, and thus, increase RyR1 Ca^2+^ leak in certain muscle disorders^27–33^. On the other hand, some RyR1-related myopathy mutations were reported to affect RyR1 channel conductance, ligand (Ca^2+^, ATP) regulation or expression of the channel^21, 34, 35^.

Diagnosis of myopathies traditionally relies on the histological findings from a muscle biopsy^36^, but recently the diagnostic procedure is enhanced by the sequencing of the entire RyR1 gene. Owing to this technical development, the number of newly discovered mutations is increasing^37–39^. Both dominant and recessive forms of RyR1-related myopathies have been reported, with the recessive forms being often associated with a more severe clinical presentation^40, 41^. An example for such a mutation is T4706M. Despite the low incidence rate of these recessive mutations, their medical relevance may be significant, because some heterozygous cases occur together with epigenetic allele silencing of the wild type allele resulting in a pseudo-recessive phenotype, including severe facial and proximal weakness, scoliosis, opthalmoplegia and respiratory impairment^42, 43^. Homozygous T4709M knock-in RyR1 mice (equivalent to the T4706M in human RyR1) were generated and examined previously^42^. Homozygous TM mice exhibit mild muscle weakness, kyphosis, enhanced isoflurane sensitivity and reduced RyR1 expression (~ 30% reduction). Compound heterozygous mice with one TM allele and one frameshift (null) allele express a markedly lower level of RyR1 channels (~ 80% reduction) and exhibit an even more severe (post-natal lethal) myopathy. Importantly, both models are expected to result in only homotypic TM/TM channels, with a higher level of overall channel expression in muscle of homozygous TM/TM mice^42^.

TM mutation is located adjacent to the RyR1 caffeine binding site^44^. Prior studies conducted on recombinant RyR1 channels expressed and purified from Hek293 cells found that the homotypic TM RyR1 channels exhibited a normal low open probability in low Ca^2+^, but an increased sensitivity to activation by caffeine. However, the function of endogenous homotypic TM channels in skeletal muscle and the degree to which TM channel dysfunction contributes to muscle dysfunction is unclear^42, 43^. This question of homotypic T4706M RyR’s functionality is particularly important because reactivation of the wt silenced allele using DNA methyltransferase inhibitors appears to be a promising future therapeutic option to improve RyR1 expression, but improvement in muscle function can only be expected if RyR1 function is adequate^43^.

As homozygous TM/TM mice (TM) express a higher level of homotypic RyR1 channels, purified RyR1 channels from skeletal muscle of wt and homozygous TM mice in order to directly evaluate the impact of the T4706M mutation on RyR1 channel function at the single-channel level. Here, we characterized the unitary conductance, ligand (Ca^2+^, ATP, and Mg^2+^) sensitivity, and biophysical and pharmacological properties of single wt and homotypic T4709M RyR1 channels reconstituted in planar lipid bilayers.

## Materials and methods

The methods were conducted in accordance with the ARRIVE guidelines. All methods were performed in accordance with the relevant guidelines and regulations. All animal studies were designed to minimize animal suffering and were approved by the University Committee on Animal Resources at the University of Rochester (UCAR2006-114E).

### Materials

Phospholipids were obtained from Avanti Polar Lipids, Inc. (Alabaster, AL). All other chemicals, if not specified, were purchased from Sigma-Aldrich (St. Louis, MO).

### Ryanodine receptor purification

13 g of skeletal muscle was collected from wild type (wt) and genetically modified homozygous mice carrying the patient-relevant T4709M mutation in RyR1 (equivalent to T4706M in human RyR1) (n = 10 for each genotype)^42^. Mice were euthanized by CO_2_ anaesthesia followed by cervical dislocation. Each step of the purification protocol was performed on ice or at 4 °C in the presence of protease inhibitors (pefabloc SC, aprotinin, leupeptin, benzamidine, pepstatin A and calpain inhibitor). The muscle samples pooled from 10 mice and were homogenised in: 100 mM NaCl, 20 mM EGTA, 20 mM Na-HEPES (pH = 7.5). Cell debris was pelleted at 3500 × *g*, for 35 min by using a laboratory centrifuge. SR microsomes were isolated by using differential centrifugation as follows. Crude microsomes were collected from the supernatant by centrifugation in a Ti45 rotor at 40,000 × *g*, for 30 min. To remove the actomyosin contamination the pellet was resuspended in: 600 mM KCl, 10 mM K-PIPES, 250 mM sucrose, 1 mM EGTA, 0.9 mM CaCl_2_ (pH = 7.0) and incubated for 1 h. Then, the microsomes were collected by centrifugation at 109,000 × *g* for 30 min, and the pellet was resuspended in: 300 mM sucrose, 10 mM K-PIPES (pH = 7.0), snap-frozen in liquid nitrogen, and stored at – 70 °C until the solubilisation step.

Microsomes were allowed to melt on ice and solubilized in: 1% CHAPS, 1 M NaCl, 100 μM EGTA, 150 μM CaCl_2_, 5 mM AMP, 0.45% phosphatidylcholine, 20 mM Na-PIPES (pH = 7.2) for 2 h. The samples were loaded onto a 10–28% linear sucrose gradient and centrifuged overnight at 90,000 × g in an SW27 rotor. RyR-containing fractions of the gradient were snap-frozen in liquid nitrogen and stored at − 70 °C in small aliquots^45–47^.

SDS-PAGE was used to verify the purified samples. 30 μl of each fraction (~ 400 μl) of the sucrose gradient was loaded in each well of a 10% linear gel. After electrophoresis, gels were stained with Coomassie Blue^6^. Images were converted to black and white and were not further processed. Full length gels are presented.

### RyR reconstitution and single-channel current recording

Voltage-clamp measurements were performed on purified RyR1 channels incorporated into artificial planar lipid bilayers. Bilayers were formed across an aperture with a diameter of 200 μm drilled in the wall of a Delrin cap (Warner Instruments, Hamden, CT), which separated two chambers. The chambers were filled with a recording solution containing: 250 mM KCl, 50 μM CaCl_2_, 10 mM HEPES (pH = 7.2). Transmembrane voltages were reference to ground (trans-to-cis), allowing ionic currents of physiological direction through open RyR channels. The free [Ca^2+^] of the recording medium was adjusted using an EGTA stock solution.

The lipid mixture contained phosphatidylethanolamine, phosphatidylserine, and phosphatidylcholine (Avanti Polar Lipids, Alabaster, AL) in a ratio of 5:4:1 was dissolved in n-decane at a final concentration of 20 mg/mL. The bilayer currents were recorded in voltage-clamp mode using an AxoPatch 200 amplifier and pCLAMP 6.03 (Axon Instruments, Sunnyvale, CA) software. The holding potentials were as indicated in the text. The currents were filtered at 1 kHz through an eight-pole low-pass Bessel filter and digitized at 3 kHz^45–47^.

### Statistics

Open probabilities (P_o_) were determined using the pClamp software suite (Molecular Devices, Sunnyvale, CA). Statistical analyses were performed in Origin 7.0 (OriginLab, Northampton, MA) and in Excel (Microsoft, Redmond, WA). Results were expressed as mean ± standard error (SE). Relative P_o_ data were calculated by normalizing each data point to their own control. Statistical significance of differences was evaluated by using the independent two-sample t-test. Differences were considered significant when p was less than 0.05 (marked by *). The number of observations is displayed in the figure.

## Results and discussion

**Figure 1:**
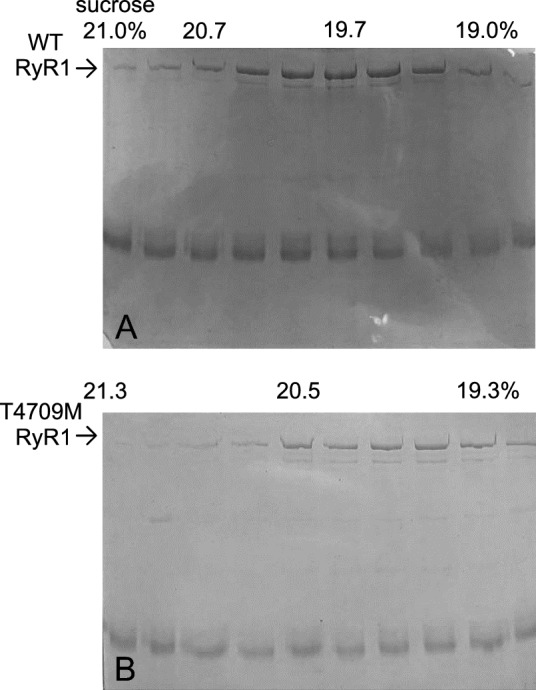
SDS-PAGE of RyR1 samples purified from skeletal muscle tissue. (**A**) Samples of different sucrose gradient fractions of a RyR1 preparation from wt mice. The arrow indicates the band of the RyR1 protein. Sucrose concentrations (%) were as indicated. (**B**) Samples of a RyR1 preparation as in (**A**) from a homozygous T4709M RyR1 mice.

Skeletal muscle was collected from wt and homozygous T4709M (TM) RyR1 knock-in mice and RyRs were purified from the tissue using sucrose gradient ultracentrifugation. The resulting samples were verified for RyR1 content using SDS-PAGE. Small volumes of 10 different fractions, collected between the 18 and 21% sucrose concentration range were loaded on a gel and stained with Coomassie blue after electrophoresis. Consistent with prior reports, the RyR1 expression was lower in the case of TM muscle, but no evidence for fragmentation of the protein was found (Fig. 1).

**Figure 2:**
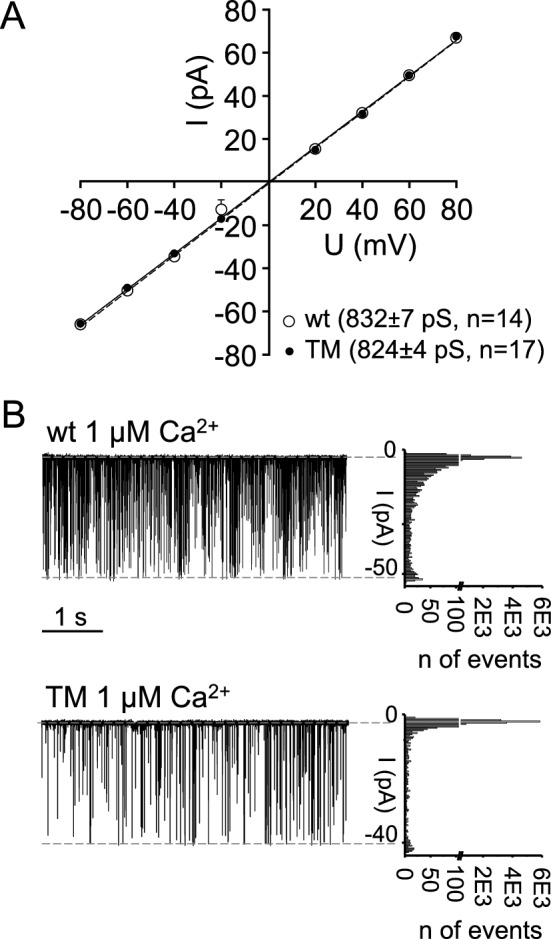
Biophysical properties of RyR1 channel currents. (**A**) Current–voltage relationships of wt and T4709M single RyR1s. Average (± SE) wt and T4709M RyR1 channel slope-conductances are indicated in the right corner. (**B**) Representative amplitude histograms at -60 mV of wt and T4709M RyR1s.

RyR channels from sucrose fractions ~ 19–21% were reconstituted in planar lipid bilayers. The single-channel conductance in 250 mM KCl recording solution not significantly different between the two groups: 832 ± 6.9 pS for wt channels and 824 ± 4.1 pS for TM channels (Fig. 2A). Current transitions, partial openings of lower amplitude (i.e. subconductive states) were not observed for either wt or TM channels, as demonstrated by current amplitude histograms shown in Fig. 2B.

**Figure 3:**
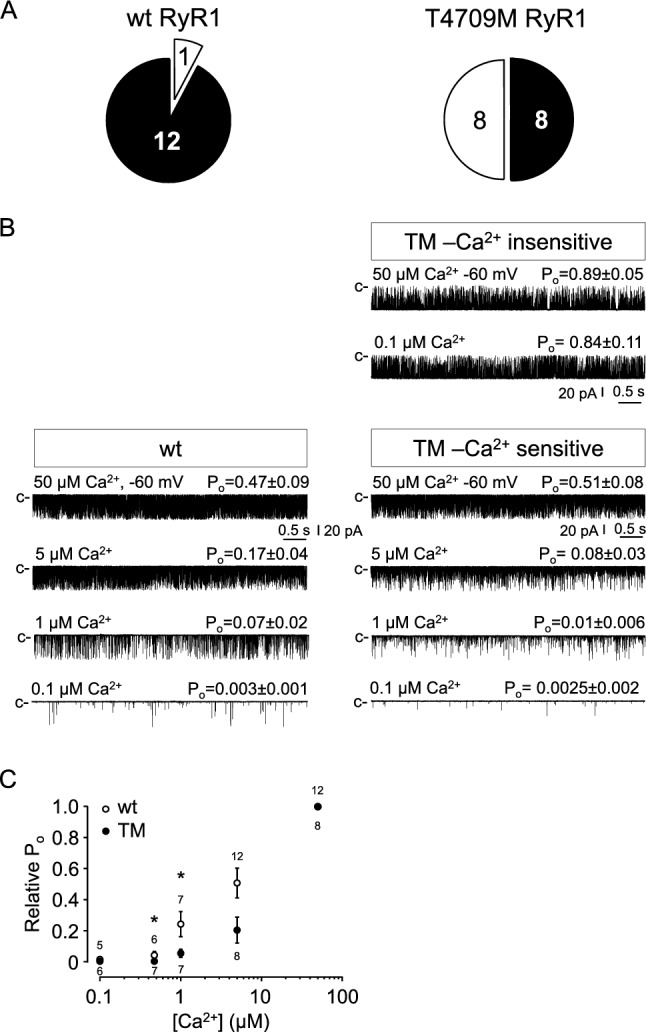
Ca^2+^ dependence of wt and T4709M RyR1 channel activity. (**A**) Pie charts summarizing the proportion of wt (left) and T4709M (right) RyR channels that responded to changes in cytosolic [Ca^2+^] (black). (**B**) Representative single channel current traces of wt and T4709M RyR1 channels at high (50 μM) and low (100 nM) cytosolic [Ca^2+^]. The closed state of the channel is marked by `c`. Downward deflections correspond to channel openings. Average open probabilities (P_o_) ± SE are indicated above each trace. (**C**) Relative RyR1 channel open probabilities (P_o_) (mean ± SE) plotted as a function of cytosolic [Ca^2+^]. P_o_s are expressed relative to control, recorded at 50 μM Ca^2+^.

As Ca^2+^ is one of the most important cytoplasmic ligands of RyRs, we compared the Ca^2+^ sensitivity of wt and TM channels. Based on this feature, TM channels clearly divided into two distinct populations. When the [Ca^2+^] on the cytoplasmic face of the channel was reduced from 50 μM, the open probability (P_o_) decreased in only 8 out of 16 TM channels (Fig. 3A), while the high P_O_ of the other 8 TM channels was not reduced after lowering cytoplasmic [Ca^2+^], which is an extraordinarily high proportion of channels. In contrast, the high P_O_ value of 12 out of 13 wt channels were reduced following reduction of cytoplasmic Ca^2+^, as is typical in our hands (i.e. ~ 10% Ca^2+^ unresponsive channels) (Fig. 3A). Representative current recordings from Ca^2+^ insensitive (or “reluctant”) and Ca^2+^ sensitive TM RyR1 channels, as well as a typical Ca^2+^ sensitive wt RyR1 in the presence of various cytoplasmic [Ca^2+^]s are shown in Fig. 3B. Comparing the Ca^2+^ sensitive TM population to wt revealed that the maximal channel activity measured at 50 μM Ca^2+^ was not significantly different between the two groups (P_o_ = 0.55 ± 0.09 vs. 0.51 ± 0.12). We compared the Ca^2+^ dependence of the Ca^2+^ sensitive channels in 5; 1; 0.5; and 0.1 μM cytoplasmic [Ca^2+^] (Fig. 3B) and found that the P_o_ of Ca^2+^ sensitive TM channels at 0.5 and 1 μM Ca^2+^ were significantly reduced compared to that of wt channels (*p* = 0.047 and 0.016), but P_o_ values were not significantly different at 100 nM (resting) Ca^2+^ (*p* = 0.8) (Fig. 3C). These data agree with earlier data obtained in Q1970fsX16 + A4329D myopathy-mutant channels ^34^. The average P_o_ of reluctant channels at 50 μM Ca^2+^ was substantially higher (0.89 ± 0.05) than that of their Ca^2+^ sensitive counterparts (0.55 ± 0.09), whereas their average P_o_ did not decrease appreciably when the [Ca^2+^] was reduced to 500 nM (0.84 ± 0.11). It should be noted that we have not previously observed a similarly high proportion of reluctant channels in prior RyR1 preparations. This unusually high proportion of Ca^2+^-insensitive, high P_O_ mutant channels would be expected to exhibit a “leaky” phenotype in spite of the absence of a significant proportion of subconduction states as is typically observed following FKBP dissociation. The reasons for the loss of Ca^2+^ regulation in this subpopulation of TM RyR1 channels is unclear, but could reflect either primary (conformational) and/or secondary (posttranslational) consequences of the mutation.

Kushnir et al. reported that the TM mutation is located adjacent to the caffeine binding site and they used H^3^ Ryanodine-binding and single channel bilayer recordings of recombinant TM channels expressed in HEK cells to conclude that TM channels exhibit a normal low P_o_ in low Ca^2+^ concentrations but an increased sensitivity to activation by caffeine (and thus, likely increased Ca^2+^ sensitivity)^26, 44^. Our results on TM/TM channels isolated from native skeletal muscle tissue are quite different or at least our observation of two different classes of channels (one with lower Ca^2+^ sensitivity and one with high P_o_ at all Ca^2+^ concentrations) is different. This suggests that it is important to assess the function of RyR1 disease mutant channels purified from native skeletal muscle as their function can be influenced by numerous muscle-specific factors (e.g. post-translational modifications, binding proteins, luminal factors, etc.).

**Figure 4:**
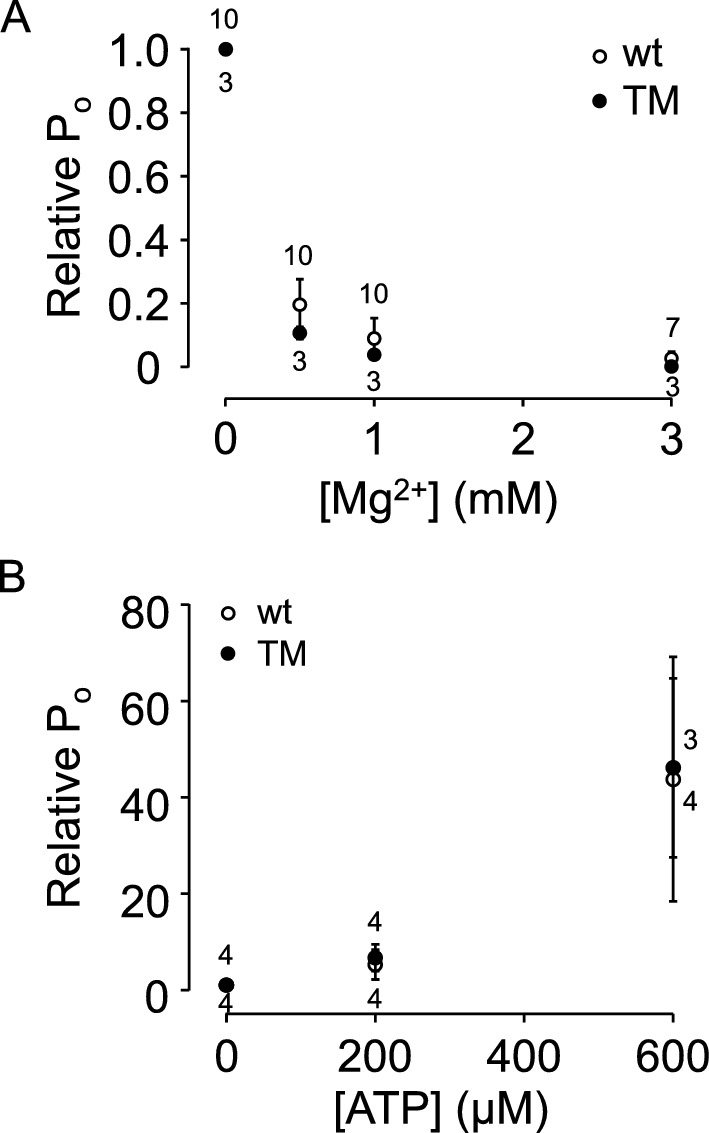
The effect of Mg^2+^- and ATP on the activity of wt and T4709M RyR1 channels. (**A**) Mean ± SE of P_o_ of wt and T4709M RyR1 channels (50 μM cytosolic Ca^2+^) at different cytosolic Mg^2+^ concentrations normalized to control P_o_ obtained at 0 μM Mg^2+^. (**B**) Mean ± SE of P_o_ of wt and T4709M RyR1 channels (0 mM cytosolic Mg^2+^ and 100 nM cytosolic Ca^2+^) at different ATP concentrations normalized to control P_o_ obtained at 0 μM ATP.

The Mg^2+^ and ATP sensitivity of wt and TM RyR1s were also tested. In the presence of 50 µM cytoplasmic [Ca^2+^], Mg^2+^ was added to the cytosolic face of the channels at increasing concentrations. The concentration-dependent inhibition of RyR1 activity (P_O_) by cytoplasmic Mg^2+^ not statistically different between wt and TM channels (Fig. 4A). Similarly, cytoplasmic ATP similarly activated both wt and TM RyR1 channels in a similar concentration dependent manner in the presence of 100 nM cytoplasmic Ca^2+^ in the absence of Mg^2+^ (Fig. 4B).

**Figure 5:**
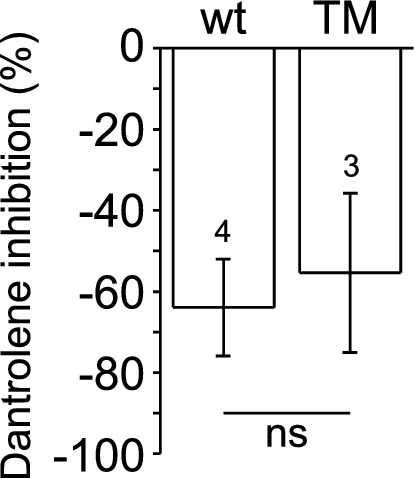
The effect of dantrolene on wt and T4709M RyR1 channels. Control single channel currents of wt and T4709M RyR1 channels were recorded in the presence of 50 μM Ca^2+^, 1 mM ATP and 3 mM Mg^2+^. The channels were then treated with 10 μM dantrolene. Data are expressed as [(P_o dantrolene_/P_o control_) − 1] × 100.

As the T4706M mutation is associated with increased susceptibility for malignant hyperthermia susceptibility^41^, we tested whether TM channels are sensitive to inhibition by dantrolene, which is the only drug used to treat a malignant hyperthermia crisis. Because dantrolene requires Mg^2+^ and ATP to inhibit the RyR1 channels in bilayers, we tested the effect of dantrolene in the presence of 1 mM ATP and 3 mM Mg^2+^ in the cytoplasmic compartment of the recording chamber^48, 49^. We found that a therapeutically relevant concentration of dantrolene (10 µM) produced a similar, significant (~ 60%) reduction in channel activity (P_O_) for both wt and TM channels (Fig. 5), demonstrating that TM channels retain dantrolene-sensitivity. It should be noted that while the Ca^2+^ sensitivity of the RyR1 channels evaluated in this series of experiments was not directly tested, based on the high P_o_ value of the TM channels in 50 µM Ca^2+^ (0.93 ± 0.03), these RyR1 channels most likely represented Ca^2+^ insensitive “reluctant” channels.

## Conclusion

In this study, we identified two functionally distinct populations of T4706M RyRs. One group exhibited high baseline P_o_ that was not reduced following a reduction in cytoplasmic Ca^2+^ to physiological levels observed in resting skeletal muscle (e.g. 100 nM), which would be expected to result in a high level of RyR1-dependent SR Ca^2+^ leak. While the other population of TM RyR1 channels exhibited a normal P_O_ at 100 nM cytoplasmic Ca^2+^_,_ these channels showed lower responsiveness to moderate elevations in cytoplasmic Ca^2+^ (0.5–10 μM) that occur during muscle excitation. Our results suggest that Ca^2+^-insensitive, high P_O_ (i.e. reluctant) channels will enhance resting SR Ca^2+^ leak and potentially deplete SR Ca^2+^, which would reduce the driving force for Ca^2+^-release and contribute to T4706M-linked myopathy. In addition, the reduced Ca^2+^-sensitivity a subpopulation of TM RyR1 channels may also participate in the pathomechanism of muscle weakness by since these channels would be less activated by cytoplasmic Ca^2+^ during muscle excitation. We must note that functional dichotomy of homotypic TM channels may reflect either primary conformational or secondary post-translational (e.g. Ca^2+^-dependent modifications) mechanisms. Although, our description of two functionally distinct classes of RyR1 channels in TM muscle falls short of determining their relative role in disease pathogenesis, these results should be taken into consideration in the development of new therapeutic interventions for RyR1-related myopathy.

## Data Availability

All data obtained or analyzed during this study are included in this published article.

## References

[1] Fill M, Copello JA (2002). Ryanodine receptor calcium release channels. Physiol. Rev..

[2] Calderón JC, Bolaños P, Caputo C (2014). The excitation–contraction coupling mechanism in skeletal muscle. Biophys. Rev..

[3] Smith JS, Coronado R, Meissner G (1985). Sarcoplasmic reticulum contains adenine nucleotide-activated calcium channels. Nature.

[4] Smith JS, Coronado R, Meissner G (1986). Single channel measurements of the calcium release channel from skeletal muscle sarcoplasmic reticulum. Activation by Ca2+ and ATP and modulation by Mg2+. J. Gen. Physiol..

[5] Smith JS, Imagawa T, Ma J, Fill M, Campbell KP, Coronado R (1988). Purified ryanodine receptor from rabbit skeletal muscle is the calcium-release channel of sarcoplasmic reticulum. J. Gen. Physiol..

[6] Sárközi S, Szegedi C, Szentesi P, Csernoch L, Kovács L, Jóna I (2000). Regulation of the rat sarcoplasmic reticulum calcium release channel by calcium. J. Muscle Res. Cell Motil..

[7] Jóna I, Szegedi C, Sárközi S, Szentesi P, Csernoch L, Kovács L (2001). Altered inhibition of the rat skeletal ryanodine receptor/calcium release channel by magnesium in the presence of ATP. Pflügers Arch. Eur. J. Physiol..

[8] Laver DR (2018). Regulation of the RyR channel gating by Ca2+ and Mg2. Biophys. Rev..

[9] Laver DR, Lenz GKE, Lamb GD (2001). Regulation of the calcium release channel from rabbit skeletal muscle by the nucleotides ATP, AMP, IMP and adenosine. J. Physiol..

[10] Ebashi S, Endo M (1968). Calcium and muscle contraction. Prog. Biophys. Mol. Biol..

[11] Endo M (2009). Calcium-induced calcium release in skeletal muscle. Physiol. Rev..

[12] Belevych AE, Radwański PB, Carnes CA, Györke S (2013). ‘Ryanopathy’: Causes and manifestations of RyR2 dysfunction in heart failure. Cardiovasc. Res..

[13] Lawal TA, Todd JJ, Meilleur KG (2018). Ryanodine receptor 1-related myopathies: Diagnostic and therapeutic approaches. Neurotherapeutics.

[14] Dowling JJ, N. K. G. H. B. A. (2015) *Neuromuscular Disorders of Infancy, Childhood, and Adolescence: A clinician’s approach*, 2nd Ed. (Darras BT, Jones HR, Ryan MM, and Vivo DCD eds), Elsevier, Academic Press, San Diego

[15] Riazi S, Kraeva N, Hopkins PM (2018). Malignant hyperthermia in the post-genomics era: New perspectives on an old concept. Anesthesiology.

[16] Kolb ME, Horne ML, Martz R (1982). Dantrolene in human malignant hyperthermia a multicenter study. Anesthesiology.

[17] MacLennan DH, Phillips MS (1992). Malignant hyperthermia. Science.

[18] Flewellen EH, Nelson TE, Jones WP, Arens JF, Wagner DL (1983). Dantrolene dose response in awake man: Implications for management of malignant hyperthermia. Anesthesiology.

[19] Van Winkle WB (1976). Calcium release from skeletal muscle sarcoplasmic reticulum: Site of action of dantrolene sodium?. Science.

[20] Fruen BR, Mickelson JR, Louis CF (1997). Dantrolene inhibition of sarcoplasmic reticulum Ca2+ release by direct and specific action at skeletal muscle ryanodine receptors. J. Biol. Chem..

[21] Zhou H, Yamaguchi N, Xu L, Wang Y, Sewry C, Jungbluth H, Zorzato F, Bertini E, Muntoni F, Meissner G, Treves S (2006). Characterization of recessive RYR1 mutations in core myopathies. Hum. Mol. Genet..

[22] Zhou H, Rokach O, Feng L, Munteanu I, Mamchaoui K, Wilmshurst JM, Sewry C, Manzur AY, Pillay K, Mouly V, Duchen M, Jungbluth H, Treves S, Muntoni F (2013). RyR1 deficiency in congenital myopathies disrupts excitation-contraction coupling. Hum. Mutat..

[23] Avila G, Dirksen RT (2001). Functional effects of central core disease mutations in the cytoplasmic region of the skeletal muscle ryanodine receptor. J. Gen. Physiol..

[24] Treves S, Jungbluth H, Muntoni F, Zorzato F (2008). Congenital muscle disorders with cores: The ryanodine receptor calcium channel paradigm. Curr. Opin. Pharmacol..

[25] Ebersole JS, Levine RA (1975). Abnormal neuronal responses during evolution of a penicillin epileptic focus in cat visual cortex. J. Neurophysiol..

[26] Kushnir A, Todd JJ, Witherspoon JW, Yuan Q, Reiken S, Lin H, Munce RH, Wajsberg B, Melville Z, Clarke OB, Wedderburn-Pugh K, Wronska A, Razaqyar MS, Chrismer IC, Shelton MO, Mankodi A, Grunseich C, Tarnopolsky MA, Tanji K, Hirano M, Riazi S, Kraeva N, Voermans NC, Gruber A, Allen C, Meilleur KG, Marks AR (2020). Intracellular calcium leak as a therapeutic target for RYR1-related myopathies. Acta Neuropathol..

[27] Reiken S, Lacampagne A, Zhou H, Kherani A, Lehnart SE, Ward C, Huang F, Gaburjakova M, Gaburjakova J, Rosemblit N, Warren MS, He K, Yi G, Wang J, Burkhoff D, Vassort G, Marks AR (2003). PKA phosphorylation activates the calcium release channel (ryanodine receptor) in skeletal muscle. J. Cell Biol..

[28] Aracena P, Tang W, Hamilton SL, Hidalgo C (2005). Effects of *S* -glutathionylation and *S* -nitrosylation on calmodulin binding to triads and FKBP12 binding to type 1 calcium release channels. Antioxid. Redox Signal..

[29] Bellinger AM, Reiken S, Carlson C, Mongillo M, Liu X, Rothman L, Matecki S, Lacampagne A, Marks AR (2009). Hypernitrosylated ryanodine receptor calcium release channels are leaky in dystrophic muscle. Nat. Med..

[30] Bellinger AM, Reiken S, Dura M, Murphy PW, Deng S-X, Landry DW, Nieman D, Lehnart SE, Samaru M, LaCampagne A, Marks AR (2008). Remodeling of ryanodine receptor complex causes “leaky” channels: A molecular mechanism for decreased exercise capacity. Proc. Natl. Acad. Sci..

[31] Andersson DC, Betzenhauser MJ, Reiken S, Meli AC, Umanskaya A, Xie W, Shiomi T, Zalk R, Lacampagne A, Marks AR (2011). Ryanodine receptor oxidation causes intracellular calcium leak and muscle weakness in aging. Cell Metab..

[32] Rullman E, Andersson DC, Melin M, Reiken S, Mancini DM, Marks AR, Lund LH, Gustafsson T (2013). Modifications of skeletal muscle ryanodine receptor type 1 and exercise intolerance in heart failure. J. Hear. Lung Transplant..

[33] Dowling JJ, Arbogast S, Hur J, Nelson DD, McEvoy A, Waugh T, Marty I, Lunardi J, Brooks SV, Kuwada JY, Ferreiro A (2012). Oxidative stress and successful antioxidant treatment in models of RYR1-related myopathy. Brain.

[34] Elbaz M, Ruiz A, Bachmann C, Eckhardt J, Pelczar P, Venturi E, Lindsay C, Wilson AD, Alhussni A, Humberstone T, Pietrangelo L, Boncompagni S, Sitsapesan R, Treves S, Zorzato F (2019). Quantitative RyR1 reduction and loss of calcium sensitivity of RyR1Q1970fsX16+A4329D cause cores and loss of muscle strength. Hum. Mol. Genet..

[35] Yuan Q, Dridi H, Clarke OB, Reiken S, Melville Z, Wronska A, Kushnir A, Zalk R, Sittenfeld L, Marks AR (2021). RyR1-related myopathy mutations in ATP and calcium binding sites impair channel regulation. Acta Neuropathol. Commun..

[36] Neto OA, Moreno CD, Malfatti E, Donkervoort S, Böhm J, Guimarães JB, Foley AR, Mohassel P, Dastgir J, Bharucha-Goebel DX, Monges S, Lubieniecki F, Collins J, Medne L, Santi M, Yum S, Banwell B, Salort-Campana E, Rendu J, Fauré J, Yis U, Eymard B, Cheraud C, Schneider R, Thompson J, Lornage X, Mesrob L, Lechner D, Boland A, Deleuze J-F, Reed UC, Oliveira ASB, Biancalana V, Romero NB, Bönnemann CG, Laporte J, Zanoteli E (2017). Common and variable clinical, histological, and imaging findings of recessive RYR1-related centronuclear myopathy patients. Neuromuscul. Disord..

[37] Gonzalez-Quereda L, Rodriguez MJ, Diaz-Manera J, Alonso-Perez J, Gallardo E, Nascimento A, Ortez C, Natera-de Benito D, Olive M, Gonzalez-Mera L, Lopez de Munain A, Zulaica M, Poza JJ, Jerico I, Torne L, Riera P, Milisenda J, Sanchez A, Garrabou G, Llano I, Madruga-Garrido M, Gallano P (2020). Targeted next-generation sequencing in a large cohort of genetically undiagnosed patients with neuromuscular disorders in spain. Genes (Basel).

[38] Foo CTY, To YH, Irwanto A, Ng AY-J, Yan B, Chew STH, Liu J, Ti LK (2022). Variant landscape of the RYR1 gene based on whole genome sequencing of the Singaporean population. Sci. Rep..

[39] Bharucha-Goebel DX, Santi M, Medne L, Zukosky K, Dastgir J, Shieh PB, Winder T, Tennekoon G, Finkel RS, Dowling JJ, Monnier N, Bonnemann CG (2013). Severe congenital RYR1-associated myopathy: The expanding clinicopathologic and genetic spectrum. Neurology.

[40] Gonorazky HD, Bönnemann CG, Dowling JJ (2018). The genetics of congenital myopathies. Handb. Clin. Neurol..

[41] Colombo I, Scoto M, Manzur AY, Robb SA, Maggi L, Gowda V, Cullup T, Yau M, Phadke R, Sewry C, Jungbluth H, Muntoni F (2015). Congenital myopathies: Natural history of a large pediatric cohort. Neurology.

[42] Brennan S, Garcia-Castañeda M, Michelucci A, Sabha N, Malik S, Groom L, Wei LaPierre L, Dowling JJ, Dirksen RT (2019). Mouse model of severe recessive RYR1-related myopathy. Hum. Mol. Genet..

[43] Zhou H, Brockington M, Jungbluth H, Monk D, Stanier P, Sewry CA, Moore GE, Muntoni F (2006). Epigenetic allele silencing unveils recessive RYR1 mutations in core myopathies. Am. J. Hum. Genet..

[44] des Georges A, Clarke OB, Zalk R, Yuan Q, Condon KJ, Grassucci RA, Hendrickson WA, Marks AR, Frank J (2016). Structural basis for gating and activation of RyR1. Cell.

[45] Sárközi S, Komáromi I, Jóna I, Almássy J (2017). Lanthanides report calcium sensor in the vestibule of ryanodine receptor. Biophys. J..

[46] Szigeti GP, Almássy J, Sztretye M, Dienes B, Szabó L, Szentesi P, Vassort G, Sárközi S, Csernoch L, Jóna I (2007). Alterations in the calcium homeostasis of skeletal muscle from postmyocardial infarcted rats. Pflügers Arch. Eur. J. Physiol..

[47] Geyer N, Diszházi G, Csernoch L, Jóna I, Almássy J (2015). Bile acids activate ryanodine receptors in pancreatic acinar cells via a direct allosteric mechanism. Cell Calcium.

[48] Diszházi G, Magyar ZÉ, Mótyán JA, Csernoch L, Jóna I, Nánási PP, Almássy J (2019). Dantrolene requires Mg^2+^ and ATP to inhibit the ryanodine receptor. Mol. Pharmacol..

[49] Choi RH, Koenig X, Launikonis BS (2017). Dantrolene requires Mg ^2+^ to arrest malignant hyperthermia. Proc. Natl. Acad. Sci..

